# SCD1 is associated with tumor promotion, late stage and poor survival in lung adenocarcinoma

**DOI:** 10.18632/oncotarget.9461

**Published:** 2016-05-19

**Authors:** Jun Huang, Xing-Xing Fan, Jiaxi He, Hui Pan, Run-Ze Li, Liyan Huang, Zebo Jiang, Xiao-Jun Yao, Liang Liu, Elaine Lai-Han Leung, Jian-Xing He

**Affiliations:** ^1^ State Key Laboratory of Respiratory Disease, Guangzhou Institute of Respiratory Disease, The 1st Affiliated Hospital of Guangzhou Medical University, Guangzhou, China; ^2^ State Key Laboratory of Quality Research in Chinese Medicine/Macau Institute for Applied Research in Medicine and Health, Macau University of Science and Technology, Macau (SAR), China; ^3^ Guangdong Cardiovascular Institute, Guangdong General Hospital, Guangdong Academy of Medical Sciences, Guangzhou, China

**Keywords:** lung adenocarcinoma, SCD1, EGFR, biomarker, lipid metabolism

## Abstract

The discovery of Warburg effect opens a new era in anti-cancer therapy. Aerobic glycolysis is regarded as a hallmark of cancer cells and increasing literatures indicates that metabolic changes are critical for the maintenance and progression of cancer cells. Besides aerobic glycolysis, increased fatty acid synthesis is also required for the rapid growth of cancer cells, and is considered as one of the most typical metabolic symbols of cancer either. Thus, targeting fatty acid metabolism may provide a potential avenue for the diagnosis and therapeutic treatment of cancer. In this study, we have identified Sterol-CoA desaturase-1 (SCD1) which is the rate-limiting enzyme of unsaturated fatty acid synthesis, universally and highly expressed in lung adenocarcinoma and was required for the cell proliferation, migration and invasion. Both *in vitro* and *in vivo* studies demonstrated that high expression of SCD1 remarkably enhanced the ability of tumor formation and invasion, while knockdown of SCD1 significantly repressed tumorigenesis and induced cell apoptosis. Clinical association study suggested that high expression of SCD1 is more frequently observed in late stage patients and presents poor prognosis. Taken together, our results suggested that SCD1 is a potentially novel biomarker of lung adenocarcinoma, and targeting SCD1 may represent a new anti-cancer strategy.

## INTRODUCTION

Lung cancer has the highest incidence and mortality of cancer in worldwide [1]. Although great efforts have made, the overall incidence and mortality rate is still very high and has become one of the biggest threats to human life and health [2]. Importantly, at present, the incidence and mortality rate of lung cancer in China are the highest all over the world [3]. As the main type of lung cancer, lung adenocarcinoma attracted the most attentions. However, even current surgery, radiotherapy and chemotherapy have significantly improved and extended survival time of patients with lung adenocarcinoma, but the effect of overall treatment remains undesirable [4]. For example, the positive response rate of platinum-based chemotherapy is poorly around 30 % and a median overall survival is only 10-12 months [5]. Although tyrosine kinase inhibitors (TKIs) such as gefitinib or eloritinib which are small molecular inhibitors of EGFR, acquires great clinical successes [6], clinical data showed that targeted therapy with TKIs didn't significantly prolong overall survival and unfortunately eventual acquired resistance is inevitable [7]. It is clear that single TKI treatment is not enough for treating NSCLC patients [8] Therefore, identifying new biomarkers and therapeutic targets with clinical relevance is of highly importance.

Recently, it was reported that increase in fatty acid synthesis, especially membrane lipids, greatly promotes proliferation of cancer cells and is essential for them to escape from programmed cell death [9] [10]. Suppressed lipid metabolism would lead to failure of the formation of cellular membrane and further destroy membrane fluidity and other physical and chemical properties, and resulting in signal transduction [11] and gene expression abnormalities [12]. Therefore, inhibiting lipid metabolism in cancer cells would be a promising strategy for anti-cancer therapy.

In this study, we focused on investigation of stearoyl-CoA desaturase-1 (SCD1) which is a critical regulator of energy metabolism and catalyzes the synthesis of monounsaturated fats, on 95 NSCLC clinical tumor samples and explored the correlation between SCD1and clinical indicators, as well as its biological effect using *in vitro* and *in vivo* lung cancer model.

## RESULTS

### SCD1 is highly expressed in lung adenocarcinoma than its adjacent normal tissue

It has been known from a report of RNAi pool screening that knockdown of SCD1 induced significant level of apoptosis in cancer cells [13]. To explore its role in cancer more comprehensively, here, we investigated the expression levels of SCD1 in clinical lung tumors and further investigated its biological role in cancer using cell model and mouse xenograft model.

**Figure 1:**
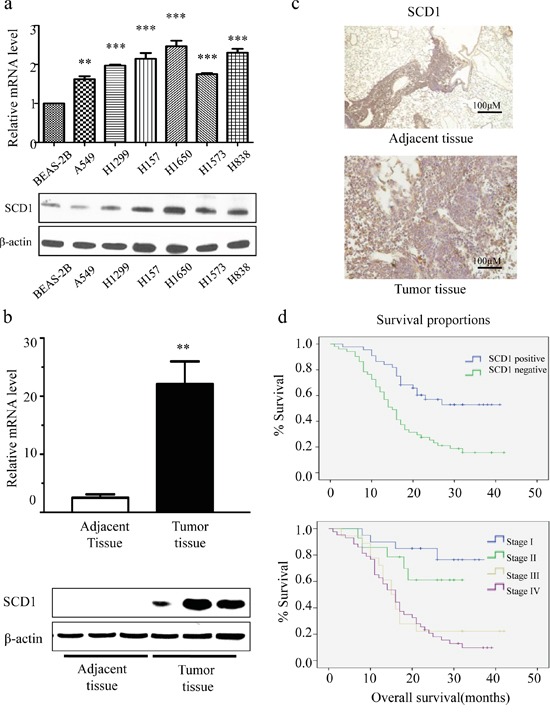
SCD1 is highly expressed in lung adenocarcinoma cells and is associated with patient survival time **a.** Compared with normal lung epithelial cell, the level of SCD1 is relatively high in NSCLC cell lines. **b.** In tumor tissue, consistent result was observed. SCD1 is much highly expressed in tumor than in adjacent normal tissue. **c.** SCD1 is universally highly expressed in lung adenocarcinoma. **d.** The expression level of SCD1 is correlated with TNM stage classification and prognosis.

Firstly, we investigated the expression status of SCD1 in 96 lung adenocarcinoma from Chinese NSCLC patients and cell lines. We compared the mRNA level and protein levels of SCD1 in normal lung epithelial cell line (BEAS-2B) with 6 different lung adenocarcinoma cell lines (A549, H1299, H1650, H157, H1573, and H838) using quantitatively real-time PCR and Western blot. Interestingly, it showed that both in RNA and protein level, SCD1 was much highly expressed in 6 lung adenocarcinoma cell lines than that of normal cells (Figure 1a). Among which, H1650 cell line has the highest expression level of SCD1. We then further validated these results in 9 pairs of lung adenocarcinoma and their adjacent tissues. Consistently, no matter in RNA level or protein level, SCD1 was much higher expressed in tumor tissue than that in adjacent tissues (Figure 1b).

Next, we further investigated whether SCD1 is universally high expressed in a larger cohort of lung adenocarcinoma, we randomly selected 95 cases of lung adenocarcinoma tissue and 48 adjacent tissues of patients to detect the expression level of SCD1. Among all samples, there are 53.7% (51/95) lung adenocarcinoma tissue highly expressed SCD1, while in adjacent tissue, only 27.1% (13/48) was considered as highly expressing SCD1 (Figure 1c). These results suggested that SCD1 universally higher expressed in lung adenocarcinoma patients.

### SCD1 expression is associated with gender, clinical stage classification and prognosis

To clarify the role of SCD1 played in lung cancer tumorigenesis and progression, we analyzed the association of SCD1 with patient's clinical and pathological data. Firstly, the relation between patient's gender and SCD1 was studied. The rate of high SCD1 patients in male and female is 61.7% (37/60) and 40.0% (14/35) respectively (Table 1). By chi square analysis of this 95 NSCLC patient cohort, SCD1 positive expression is associated with male (P<0.05), indicating that SCD1 is more universally and highly expressed in male patients. Moreover, we also investigated the association between SCD1 and clinical TNM stage classification. Attractively, the expression level of SCD1 is gradually increased with the progress of tumor stages. The rate of high SCD1 in lung adenocarcinoma stage I patients is 38.5% (5/13), while the positive rate of stage IV patients increased to 74.4% (32/43) (Table 2). Therefore, positive SCD1 increased with the progress of TNM staging in lung cancer. Based on TNM stage classification, the correlation between prognosis and SCD1 was further investigated (Table 3 and Figure 1d). The 3-year survival rate of patients is gradually decreased in patients group with high SCD1 expression. For example, in stage I, the 3-year survival rate is 76.5%. However, it drops to 61.1% in stage II, and becomes 22.2% in stage III. In stage IV, it is even less than 10%. From this part of clinical study, it is demonstrated that the expression of SCD1 is closely related with patient's gender and cancer progress and the prognosis of patients.

**Table 1:** Correlation analysis of gender with SCD1 expression in a cohort of 95 NSCLC patients

Gender	SCD1 negative % (patients count)	SCD1 positive % (patients count)	Total patients count
**Female**	**60.0% (21)**	**40.0% (14)**	**35**
**Male**	**38.3% (23)**	**61.7% (37)**	**60**

**Table 2:** Correlation analysis of TNM classification with SCD1 expression in a cohort of 95 NSCLC patients

TNM classification	SCD1 negative % (patients count)	SCD1 positive % (patients count)	Total patients count
**I a**	**61.5% (8)**	**38.5% (5)**	**13**
**I b**	**57.1% (4)**	**42.9% (3)**	**7**
**II a**	**50.0% (6)**	**50.0% (6)**	**12**
**II b**	**50.0% (1)**	**50.0% (1)**	**2**
**III a**	**85.7% (12)**	**14.3% (2)**	**14**
**III b**	**50.0% (2)**	**50.0% (2)**	**4**
**IV**	**25.6% (11)**	**74.4% (32)**	**43**
**Total percent**	**46.3% (44)**	**53.7% (51)**	**95**

**Table 3:** SCD1 positive expression level is associated with poor 3-years-survival in a 95 NSCLC patients cohort

SCD1	median	3-year-survival(%)
**Negative**	**21**	**52.9**
**Positive**	**15**	**29.4**

### SCD1 promotes tumorigenesis

**Figure 2:**
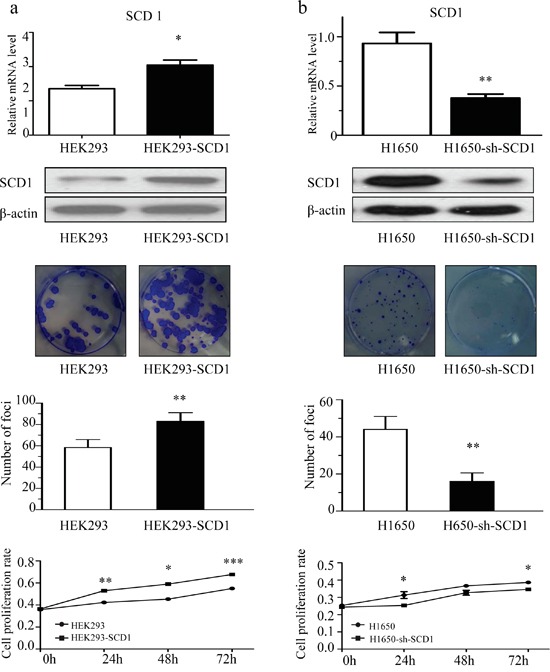
SCD1 promoted cell colony formation and proliferation **a.** Overexpression of SCD1 in HEK293 significantly promoted the colony formation and growth of cells. **b.** Knockdown of SCD1 in H1650 cells showed remarkably inhibition on cell growth.

Since the expression of SCD1 is related with cancer progress according to the clinical data, we hypothesized that SCD1 is critical for the malignant behaviors of lung adenocarcinoma. To assess the role of SCD1 in regulating cell proliferation and colony formation assay. Firstly, we transfected and overexpressed SCD1 in HEK 293 cells, and pcDNA3 was used as vehicle control. After 3 week, the number of clones was counted. The number of clones in SCD1 overexpressing group was much more than that of control group (Figure 2a). Oppositely, since SCD1 is most highly expressed in gefitinib-resistant NSCLC H1650, we established a stable SCD1 knockdown cell line using sh-SCD1 in H1650 cells and colony formation were performed. Compared with control group, sh-SCD1 knockdown group has much less and smaller clones (Figure 2b), suggesting that SCD1 is associated with malignant behaviors of cancer cells and it can remarkably promote the growth of cancer.

Furthermore, we have quantified and compared the number of apoptotic cells in H1650-sh-SCD1 and H1650 cells. Interestingly, the percentage of apoptotic cells in SCD1 knockdown group is 15.6% on average, whereas that of control group is only 3.4%, indicating that knockdown of SCD1 can promote cell apoptosis in H1650 (Supplementary Figure 1a). Moreover, the results of western blot showed that down-regulation of SCD1 increased the activation of caspase-3/9 and the expression of pro-apoptotic protein BAX, while anti-apoptotic protein BCL-2 decreased (Supplementary Figure 1a).

### SCD1 promotes cell migration and invasion

**Figure 3:**
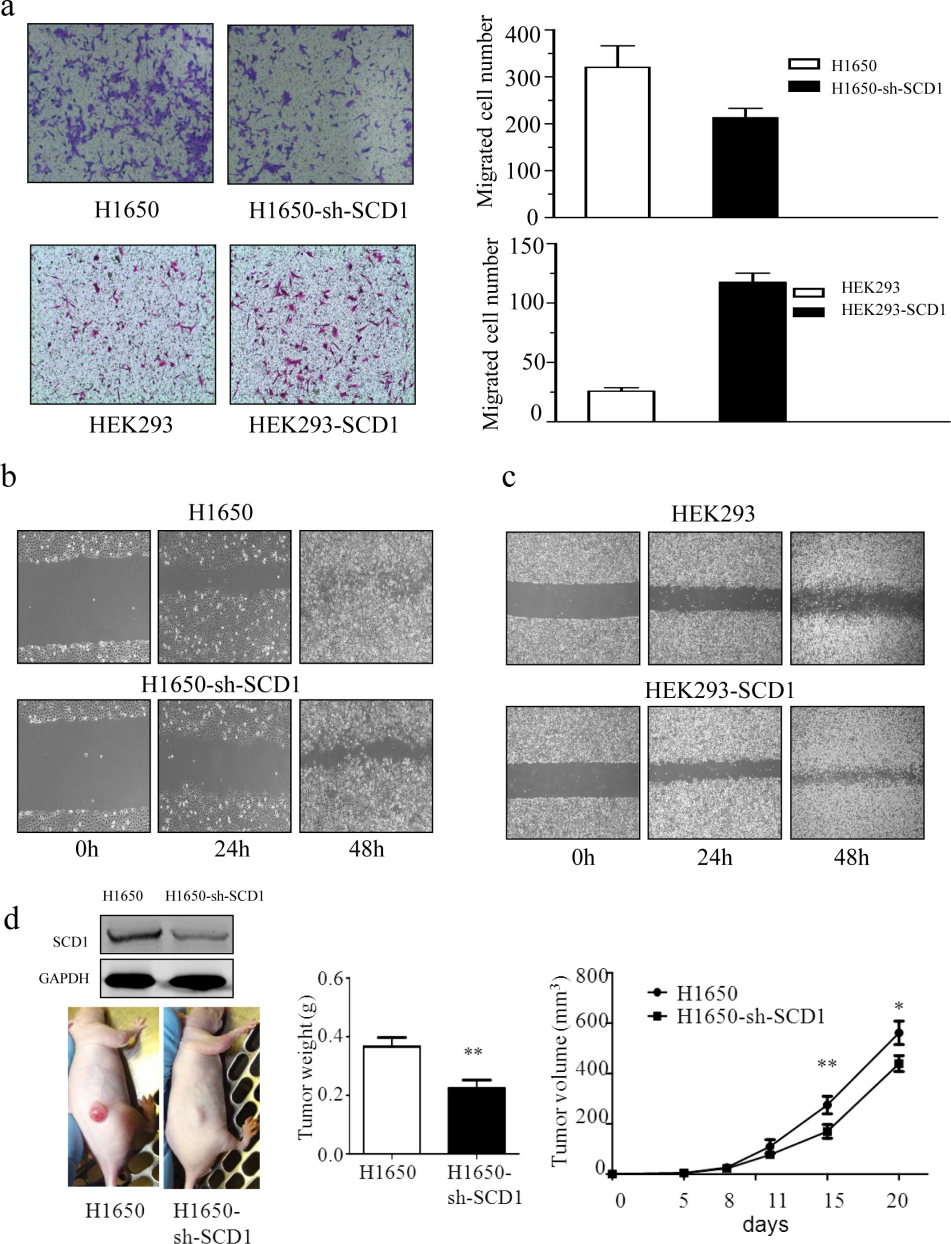
SCD1 is positively associated with the cellular ability of migration and invasion **a.** Trans-well assay indicated that knockdown of SCD1 in H1650 significantly suppressed the invasive ability of cells, whereas overexpression of SCD1 in HEK293 remarkably enhance that ability of cells. **b.** and **c.** The exrepssion level of SCD1 positively correlated with cell migration. **d.** *In vivo* study showed that tumor weight and size is much lighter and smaller in SCD1 knockdown H1650 cells.

The capacity of cell migration and invasion is another important malignant behavior of cancer progression. To determine whether SCD1 promotes cell invasion and migration, we firstly investigated the effect of SCD1 using matrigel transwell and wound healing assay. Knockdown of SCD1 in H1650 significantly reduced the cell ability of invasion and migration; the number of trans-membrane cells decreased to 113 ± 22, whereas that of H1650 cells is more than 300 (Figure 3a). Moreover, we overexpressed SCD1 in HEK293 cell and investigated with transwell and wound healing assay. Consistently, high expression of SCD1 contributes to promotion of cell invasion and migration (Figure 3b).

The result of wound healing assay also demonstrated that knockdown of SCD1 in H1650 greatly inhibited the cell migration, whereas overexpression it in HEK293 significantly accelerated cell migration (Figure 3c). Therefore, from this part of results, it demonstrated that SCD1 plays role in remarkably enhancing cell migration and invasion.

### SCD1 enhances tumorigenesis and cancer progression *in vivo*

To determine whether SCD1 can accelerate the tumor growth *in vivo*, we implanted H1650 and H1650-sh-SCD1 expressing cancer cells in nude mice. The tumor volume was measured every 3-5 days, and tumors were harvested and their tumor weight was measured at day 21. Consistent with our *in vitro* results, SCD1 significantly promoted tumor growth. The tumor weight and volume in H1650-sh-SCD1 group are much lighter and smaller than that of the control group. The average of tumors weight at H1650-SCD1 is 0.516 ± 0.031 g and that of H1650 group is 0.366±0.068 g (Figure 3d). These results indicated that SCD1 plays an important role in promoting tumor growth *in vivo*.

## DISCUSSION

In last decade, technological advance, like next-generation sequencing, makes it possible to analyze in-depth of genomes and signaling pathways. Based on these technologies and investigations, lung cancer has been defined as a group of genetic diseases with cellular heterogeneity [14]. Many lung cancer driver gene mutations have been identified, such as EGFR [15], KRAS [16], ROS [17], EML4-ALK [18]. Based on these, some specific tyrosine kinase inhibitors have been developed for anti-cancer therapy and acquired great success. For example, gefitinib or elortinib, an EGFR tyrosine kinase inhibitor has been extensively used in clinical and acquired great achievement. However, the medium time for patients to develop chemo-resistance is about12 months [19]. Therefore, single target for cancer therapy seems not enough. To discover more novel and potential anti-cancer targets is required.

Metabolism in cancer cells differs from that in normal cells [20–22]. For example, the generation of energy in cancer cells predominantly depends on aerobic glycolysis which is termed ‘Warburg effect’, while normal cells is mainly supported by oxidative phosphorylation [23]. Therefore, given that cancer cells largely rely on altered metabolism to support their rapid growth, targeting cancer metabolism may provide new strategies for cancer therapy which can effectively remove cancer cells, but spare normal cells.

In our study, we have identified SCD1 as a new biomarker and anti-cancer target for lung adenocarcinoma. Mainly physiological function of SCD1 is to catalyze saturated fatty acids (SFA) into monounsaturated fatty acid (MUFA) and maintain the balance of the ratio of SFA/MUFA which would promote cellular lipid synthesis and meet the functional requirements of cells [24]. Due to this crucial function in lipid metabolism, its cancer related role has also been extensively studied. For example, SCD1 was demonstrated to be highly expressed in breast cancer [25], and high SCD1 expression is associated with shorter survival [26]. The potential underlying mechanism of the tumor promotion function of SCD1 in cancer cell may be due to the two reasons. Firstly, SCD1 is the rate-limiting enzyme of unsaturated fatty acid synthesis. For mammalian cells, membrane is composed of a higher proportion of polyunsaturated fatty acids. Therefore SCD1 is essential for the formation and stabilization of cell membranes. It has been demonstrated that unsaturated fatty acids are required for tumor cell survival [27]. Secondly, the proliferation rate of carcinoma is faster than that of normal cells, lung adenocarcinoma requires much more extra unsaturated fatty acids to address their demands on growth, which will significantly stimulate the expression and activation of SCD1 [28]. In addition, siRNA knockdown experiment also provided the direct evidence to support that cancer cells depend on SCD1 to growth [29]. Depleting SCD in this SV40 transformed cells exhibited a dramatic decrease in proliferation rate and abolition of anchorage-independent growth [30].

Moreover, the molecular mechanism of SCD1 in promoting the proliferation of cancer cells was shown to be associated with inhibition of EGFR signal pathway [31]. The inhibitor of SCD1 could remarkably impair the ligand-induced phosphorylation of EGFR, causing the inactivation of its downstream [31]. Thus, we down-regulated SCD1 in an EGFR activation mutation lung cancer cell lines H1650 and investigated the ability of cell proliferation and invasion. The results indicated that knockdown of SCD1 significantly impaired such malignant behaviors of cells *in vitro* and *in vivo*. Overexpression of SCD1 in normal cells to mimic cancer cells indicated that SCD1 is able to promote cells growth and migration. These are all indicated that SCD1 is deeply involved in the development of lung adenocarcinoma.

To connect SCD1 with clinical information, we investigated the correlation between SCD1 and clinical indicators, such as differential expression in tumor and normal tissue, patient's gender, TNM classification and prognosis. Firstly, SCD1 is universally and highly expressed in lung adenocarcinoma. Next, it is showed that SCD1 is more universally expressed in male patients. TNM classification and 3-year survival were proved to be positively related with SCD1 expression level. Higher expression level of SCD1 was observed in the later stage of cancer patients and accompanied with lower 3-year survival rate.

In sum, it is the first time to report the association between SCD1 and clinical indicators of lung adenocarcinoma. Our study has provided evidences to support the potential role of SCD1 as a biomarker and target for cancer diagnosis and therapy in lung adenocarcinoma.

## MATERIALS AND METHODS

### Cell culture

HEK293, A549, H1299, H157, H1573, H838, H1650 and BEAS-2B were purchased from ATCC. HEK293 was cultured with DMEM medium. The culture flasks of BEAS-2B were pre-coated with a mixture of 0.01 mg/ml fibronectin, 0.03 mg/ml bovine collagen type I and 0.01 mg/ml bovine serum albumin dissolved in BEBM medium (Lonza), then cultured with BEBM. Other cell lines were cultured with RPMI 1640 medium, both of which were supplemented with 10% fetal bovine serum, 100U/mL penicillin and 100 μg/mL streptomycin (Gibco). All the cells were cultivated at 37°C with 5 % CO_2_ incubator. Cells were all cultivated at 37°C with 5% CO_2_ incubator.

### Tissue specimens

NSCLC tumor specimens were collected with informed consent from 95 patients who underwent surgical resection at the Guangzhou Medical University First Affiliated Hospital, Department of Thoracic Surgery, from 2012 to 2013. Written consent statements were obtained from all patients before operation. Frozen and formalin-fixed and parafiin-embedded primary lung cancer samples were collected under the approval of Ethical Committee. Patients follow up were performed by Dr. Hui Pan who is clinician at the Guangzhou Medical University First Affiliated Hospital. The file number of the approval of the ethics committee for the investigation of human material is 2015015 which is approved on 15^th^ January 2015.

### Lentivirus transfection

To generate SCD1 expression vector, SCD1 was amplified from the normal cDNA template and inserted in frame into pcDNA3.1. The SCD1 was cloned into pCR-Blunt with Hind III and EcoR I sites. Each expression plasmid was transfected into human embryonic kidney 293T cells using Lipo2000 (Invitrogen) to generate lentivirus carrying target gene. The lentivirus was collected 72 hours after transfection and used for target cells infection. Primers and shRNA sequence were as follow. SCD1 Forward: 5′-AGCTTATGCCGGCCCACTTGCTGCAGGACG-3′; SCD1 Reverse: 5′- AATTCTCTACCTTTGATGTTCTCACCGACT -3′; shRNA-SCD1: 5′-AGCTTCTACGGCTCTTTCTGATCATT-3′.

### Reverse transcription PCR and quantitative real-time PCR

RNAs of cells were extracted by Trizol method (Invitrogen), and used for first-strand cDNA synthesis using oligo (dT) primers and RT-PCR kit (TaKaRa). Quantitative reverse transcription PCR (qRT-PCR) was conducted using Power SYBR Green PCR Master Mix (Applied Biosystems). The qRT-PCR results were analyzed by the ΔΔCt method using beta-actin as a housekeeping gene. Primers are as following. SCD1 Forward: 5′- CGATGCCCCTCTACTTGGAA-3′; SCD1Reverse: 5′- ATACTTACCCGAGCACTGGT-3′; β-actin Forward: 5′-CATGTACGTTGCTATCCAGGC-3′; β-actin Reverse: 5′-CTCCTTAATGTCACGCACGAT-3′.

### Colony formation assay

Two hundred cells per well were seeded in 6-well plates and cultured in 37°C with 5% CO_2_ condition. At the indicated times (10 days), the cells were washed with 1X PBS 3 times and fixed with 4% PFA. After that, cells were stained with crystal violet (con. The number of clones was counted by two different researchers and calculated by a senior researcher. Clone formation rate = (clone number/cell number) ×100%.

### Immunohistochemistry Test

5um thick deparaffinized tissue sections were subjected to antigen retrieval using pressure cooker for 5 minutes in 1mM EDTA buffer. Endogenous peroxidase was quenched with 3% hydrogen peroxide for 10 minutes. Blocked sections were labeled with rabbit polyclonal anti-SCD1 antibody (abcam; 0.5mg/ml, 100ul) at the concentration of 6ug/ml overnight at 4°C. Anti-rabbit HRP-labeled polymer (DAKO) was used as a secondary antibody. Color detection was performed by liquid DAB+ substrate chromogen system (DAKO). Primary antibody was omitted in control reaction. Immunohistochemical scoring of SCD1 expression was performed regarding area and grade. The criteria were defined as follows: Over 70% of the tumor cells stained positively was scored as high (3 scores); less than 70% of the tumor cells stained positively was scored as medium (2 scores); less than 30% of the tumor cells were positively was scored as low (1 score). No color was scored as 0; light yellow staining was scored as 1; yellow staining was scored as 2; dark yellow staining was scored as 3. The final score was calculated by area scores and grade scores.

### Apoptosis analysis

1 × 10^5^ cells/well were seed into 6 well-plates and cultured overnight for adhesion. After treatment, cells were harvested and stained with Annexin V and PI for 15 min at room temperature in dark. The detection of apoptotic cells was performed and analyzed by a flow cytometer (BD FACSAria III).

### Cell cycle analysis

In order to determine the distribution of cell populations in different phases of cell cycle, cells were collected and treated with 50 μl RNase I (1 mg/ml) and stained with 5 μl propidium iodide (1 mg/ml). The percentage of cells in cell cycle phases was analyzed by flow cytometer.

### Western blot

Cells were lysed by RIPA lysis buffer. Thirty μg of proteins were loaded on 10% SDS-PAGE gel for separation and transferred to nitrocellulose membranes for detection by immunoblotting. Primary anti-bodies were incubated overnight at 4°C, and second fluorescence anti-bodies were added at room temperature for 1 hour. GAPGH was used as the loading control.

### Transwell assay

A total of 2 × 10^4^ cells were added to the upper compartment of a 24-well transwell Matrigel Invasion Chamber (Corning) in serum-free DMEM. After 24 hours, invading cells were fixed, stained with crystal violet 0.1%, and counted. The number of cells were counted in 9 random fields of view at 100 x magnification and expressed as the average number per field of view.

### Wound healing assay

Cells were seeded on 6 cm dishes at 1 × 10^6^ cells/ml and cultured in medium with 10% FBS until 90% confluence, followed by starvation with serum-free medium for 12 h. The wound line was generated by sculpturing cell culture using a sterile 10 μl-pipette tip. Images of resulting culture were obtained at 0, 24, and 48h using a light microscopy with digital camera.

### Xenograft model in nude mice

The concentration of cells was adjusted to 1×107/ml in RPMI 1640 medium and 1×106 cells were injected into the backside of nude mice. The tumor volume was measured every 3-5 days and the tumor weight was acquired at 21 days. The tumour volume was calculated by the equation: volume = (width2 × length)/2.

### Statistical analysis

Statistical analysis was done by SPSS16.0 statistical software, using the two-tailed detection, *P*<0.05 was considered statistically significant. All the data were expressed as “mean ± standard deviation“ $\left(\bar{x}\pm s\right)$; two samples were compared using the student's t-test; multiple samples were compared using one-way ANOVA test. Correlation analysis was performed using Chi square test. P<0.05 thought were statistically significant.

## SUPPLEMENTARY FIGURE


