# Supplementary material for: SCD1 is associated with tumor promotion, late stage and poor survival in lung adenocarcinoma
Source: Oncotarget. 2016 May 19;7(26):39970–9. doi: 10.18632/oncotarget.9461 (PMC5129985; doi:10.18632/oncotarget.9461)
Supplement: Supplementary file 1 [file oncotarget-07-39970-s001.pdf]

SCD1 is associated with tumor promotion, late stage and poor survival in lung adenocarcinoma

SUPPLEMENTARY FIGURE

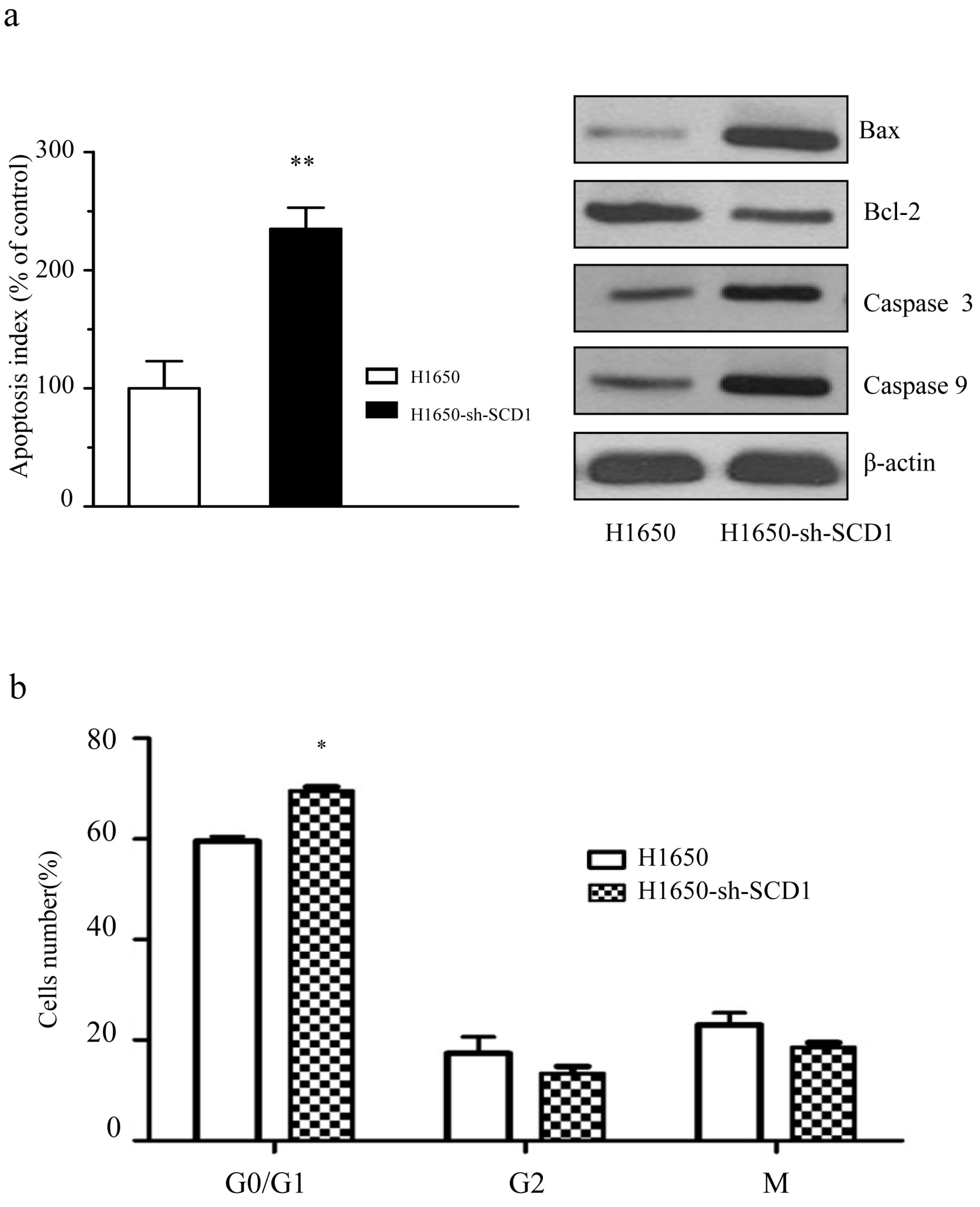

Supplementary Figure S1: a. Compared with control, the percentage of apoptotic cells largely increased in H1650-sh-SCD1 cells. Western-Blot results confirmed both: caspase 3 and 7 were activated; the ratio of BCL-2/BAX was significantly decreased. **b.** Knockdown of SCD1 caused G1 arrest in H1650.
